# Roles of Micro Ribonucleic Acids in Astrocytes After Cerebral Stroke

**DOI:** 10.3389/fncel.2022.890762

**Published:** 2022-06-09

**Authors:** Yuansheng Zhang, Li Lei, Hu Zhou, Xiaoyang Lu, Feifei Cai, Tao Li

**Affiliations:** ^1^Department of Neurosurgery, The Affiliated Hospital of Medical College, Kunming University of Science and Technology, The First People’s Hospital of Yunnan Province, Kunming, China; ^2^Translational Neurosurgery and Neurobiology, University Hospital Aachen, RWTH Aachen, Aachen, Germany; ^3^Department of Radiology, Shaoxing People’s Hospital (Shaoxing Hospital, Zhejiang University School of Medicine), Shaoxing, China

**Keywords:** cerebral stroke, micro ribonucleic acid, aquaporin 4, inflammatory response, apoptosis, autophagy, angiogenesis

## Abstract

Cerebral stroke is one of the highest-ranking causes of death and the leading cause of disability globally, particularly with an increasing incidence and prevalence in developing countries. Steadily more evidence has indicated that micro ribonucleic acids (miRNAs) have important regulatory functions in gene transcription and translation in the course of cerebral stroke. It is beyond arduous to understand the pathophysiology of cerebral stroke, due in part to the perplexity of influencing the network of the inflammatory response, brain edema, autophagy and neuronal apoptosis. The recent research shows miRNA plays a key role in regulating aquaporin 4 (AQP4), and many essential pathological processes after cerebral stroke. This article reviews the recent knowledge on how miRNA influences the inflammatory response, brain edema, infarction size, and neuronal injury after cerebral stroke. In addition, some miRNAs may serve as potential biomarkers in stroke diagnosis and therapy since the expression of some miRNAs in the blood is stable after cerebral stroke.

## Introduction

Cerebral stroke is one of the critical diseases that causes the most adult deaths and disability, comprising a major factor that makes labor loss worldwide (Wang et al., 2017; Campbell et al., 2019). Cerebral stroke is generally divided into two categories, one is ischemic stroke, with the highest incidence; the other is hemorrhagic stroke, which accounts for the smaller part but with higher mortality and severer clinical outcome (Grysiewicz et al., 2008). The pathogenesis of ischemic stroke is mainly due to the stagnation of blood flow and the resulting energy supply cut-off in the perfusion region, thereby causing damage to the neurological system, such as brain infarction, cerebral edema, and inflammatory response (Stanzione et al., 2020). For ischemic stroke, intravenous injection of recombinant tissue plasminogen activator (tPA) or interventional thrombectomy/embolectomy in time-window may save part of neuronal function (Han et al., 2018). The hemorrhagic stroke, on the other hand, is the consequence of non-traumatic blood vessel rupture, resulting in an intracranial hematoma. The surgical removal of hematoma was believed the key to improving the mortality of hemorrhagic stroke (Campbell et al., 2019). However, at present, no matter whether it is an ischemic or hemorrhagic stroke, there is no effective treatment once the neurofunctional injuries come into being. This is because there are many secondary brain injuries in the process of stroke, involving neuroinflammation, cerebral edema, hydrocephalus, blood-brain barrier (BBB) leakage, and neuronal apoptosis (Sehba et al., 2012; Wilkinson et al., 2018; Chaudhary et al., 2019). Therefore, protection and reversal of the permanent brain complications after stroke will be a breakthrough for future treatment (Sun et al., 2019). Many studies have proved that astrocytes serve an important role in stroke (Xu et al., 2020). Thus, it has been a research focus on the function of astrocytes in cerebral stroke.

Astrocytes are one type of glial cell in the central nervous system (CNS), which help to maintain the homeostasis of the brain (Poskanzer and Molofsky, 2018; Ortiz-Rodriguez and Arevalo, 2020). It has a wide range of important roles in the CNS, and there is a body of research revealing that the function of the astrocytes and neurons in the same region can be coordinated (Khakh and Deneen, 2019). Especially for patients with stroke, it can protect the nervous system and improve neurofunctional outcomes (Liu and Chopp, 2016). Astrocytes have multiple functions, including conducting a cell signal network in the brain with a gap link to transmit information molecules to achieve inter-cellular communications, participating in the secretion of neurotransmitters and neurotrophins, neurological repair and regeneration, regulating the microenvironment of the neurovascular unit, forming the BBB, helping to regulate immune function and modulate synaptic transmission (Khakh, 2019; Robinson et al., 2020; Guo et al., 2021; Patabendige et al., 2021). The processes of astrocytes can envelop the pre- and post- terminals, forming a physical barrier to restrict the diffusion of neurotransmitters from the protrusions and maintain the concentration of extracellular neurotransmitters (Liu and Chopp, 2016). Astrocytes play a direct and interactive role with neurons in synaptic transmission by regulating glutamate release [19]. Astrocytes quickly remove potassium ion accumulated by neuronal activity, absorb glutamate released during neurotransmission, convert glutamate to glutamine, and finally release it back to the presynaptic terminal. Astrocytes are also involved in the formation and integrity of the BBB together with cerebral capillary endothelial cells, basement membranes and pericytes (Armulik et al., 2010; Liu and Chopp, 2016). Many studies have shown that astrocytes have a very important position in CNS diseases, including stroke, brain trauma and neurodegenerative diseases (Pavlou et al., 2019; Sofroniew, 2020).

Non-coding ribonucleic acids (ncRNAs) are a set of RNAs conventionally regarded as non-functional but recognized functioning to regulate gene expression in a post-transcriptional manner in recent years (Heggermont and Heymans, 2012). The ncRNAs include transfer RNAs (tRNAs), ribosomal RNAs (rRNAs), long non-coding RNAs (lncRNAs) and small RNAs (sRNAs) such as microRNAs (miRNAs), circular RNAs (circRNAs) and small nuclear RNAs (snRNAs). The ncRNAs are highly expressed in the brain (Heydari et al., 2020), participating in physiological and pathophysiological processes such as cerebral ischemic injury, nerve degeneration, neurological development and plasticity (Mirzaei et al., 2018).

The miRNA is a family of short single-stranded RNAs of 19–25 nucleotide unit (Krol et al., 2010; Lu and Rothenberg, 2018). Although they are non-coding RNA molecules, it is certified that they are basic regulators of epigenetics by targeting epigenetic modification such as DNA methylation, thus changing the expression of genes (Ghoreishy et al., 2019; Saliminejad et al., 2019). They can negatively regulate genes at the mRNA level (Carthew and Sontheimer, 2009). (Most miRNAs are produced and processed by a series of intra-nuclear and extra-nuclear routes). The first step starts in the nucleus to form the primary miRNA (pri-miRNA) under the transcription of RNA polymerase 2 (Pol II). The pri-miRNA then forms the pre-miRNA which is the precursor of unmatured miRNA with a stem ring structure of 70 nucleotides under the action of ribonuclease (RNase) III Drosha, through which, the pre-miRNAs become rich. The second step takes place in the cytoplasm, where the pre-miRNAs are processed by another RNase III Dicer into double-stranded RNAs (Treiber et al., 2017). The double-strand RNA further binds to AGO protein in the cytoplasm to form an RNA-induced silencing complex (RISC). Then, the double-stranded RNA is untied, one of which is retained and the other is degraded. Finally, the mature miRNA is fully formed (Kim, 2005).

The miRNA can be used as a biomarker for specific diseases (Tiedt and Dichgans, 2018). The mature miRNAs can be transmitted directly between cells through gap links as the biosignals. Not only that, there is a miRNA called let-7 outside the cell that can regulate the gene expression through the modulation of systematic gene expression, and it has been reported to affect many pathophysiologies such as organ development, cancer genesis, and neuronal degeneration. Another example, a cluster of miRNAs including miR-29a-3p, miR-34b-3p and miR-517a-3p, is significantly decreased in the cell line of neuroblastoma (NB; Xin et al., 2017).

It is also demonstrated that miRNAs are closely related to the growth and differentiation of neurons, memory and learning functions, neurodegenerative diseases, and mental disorders (Zhang X. et al., 2019; Sun S. et al., 2020). miRNAs in astrocytes play an important role in the process of genetic transcription by combining the target DNA sequences to activate or suppress transcriptional processes. Furthermore, numerous studies reported that miRNAs play an important role in the secondary injuries after cerebral stroke, including brain edema (Zheng et al., 2017), inflammatory response (Tian et al., 2020), angiogenesis (Du et al., 2019), autophagy (Miao et al., 2020), and neuron apoptosis (Miao et al., 2020). This paper reviews part of the role of miRNAs in cerebral stroke, hoping it could provide new methods for diagnosis and treatment for the patients.

## Micro Ribonucleic Acid and Aquaporin 4

The BBB can regulate the substance exchange between the blood and the central nervous neurons (Yang et al., 2019). After stroke onset, this barrier will be damaged, and the endothelial cells that make up the tight junction of BBB will become loose, leading to swelling of astrocytes, followed by cerebral edema and a series of severe complications. It is also an important factor inducing secondary ischemic injury (Sweeney et al., 2019; Guo et al., 2021). There are usually two stages in the occurrence of cerebral edema (Mestre et al., 2020). One is the early cytotoxic stage, which is the rapid occurrence of cerebral edema within the first few minutes of the damage to the CNS. In this stage, the mechanism of ion transportation collapses, resulting in a large accumulation of intracellular ions, especially sodium and chloride ions, which eventually leads to cell swelling (Rungta et al., 2015). In the late stage, due to angiogenesis and the increase of vascular permeability, the volume of liquid in the perivascular space escalates, causing cerebral edema. However, there is no obvious boundary between the two stages (Rungta et al., 2015; Mestre et al., 2020).

The glymphatic system (GS) is a recently discovered highly organized cerebrospinal fluid (CSF) transport system in the brain (Rasmussen et al., 2018). It is responsible for metabolizing waste and expelling fluids (Lv et al., 2021). In current studies, it has been shown that in the early stage of stroke, CSF enters brain tissue through the GS, which might be the main mechanism of edema formation and ion transportation disorders (Mestre et al., 2020). There are two peaks for CSF entering the brain, one of which pours into the brain at first under the action of a concentration gradient. The second peak is that in the state of depolarization caused by ischemia or hemorrhage, CSF enters brain tissue in large quantities. AQP4 on astrocytic endfeet plays a critical role in maintaining the normal drainage function of the GS by facilitating the bulk flow of CSF in the perivascular space of penetrating arteries into the brain parenchyma, where it exchanges with the interstitial fluid (ISF) and removes the metabolites (Mestre et al., 2020). Recent *in vivo* research shows that AQP4 expression is reduced around the perihematomal edema (PHE) area after intracerebral hemorrhage. The PHE lesions in AQP4-deficient mice were significantly larger than that in the wild-type mice, which is related to poor survival outcomes (Jeon et al., 2021). This result implies that AQP4 occupies a very important position in the occurrence of cerebral edema (Nagelhus and Ottersen, 2013; Zhang J. et al., 2019; Chen et al., 2021).

Aquaporin 4 is a subtype of aquaporins (AQPs) which is also called the water channel protein (Nagelhus and Ottersen, 2013). AQP4 is mainly expressed in the brain, especially at the endfeet of astrocytes (Benarroch, 2007). In experiments on mice knocked out of AQP4, it has proved that AQP4 may be involved in CSF reabsorption, osmotic rebalance, and cerebral edema regulation (Mestre et al., 2020). AQP4 is composed of six bilayer-spanning domains and five connecting loops, forming a tetramer protein complex, each of which has its central hole (Benarroch, 2007; MacAulay, 2021). Under freeze-fracture immunogold labeling technique, it is shown that AQP4 is a microcrystalline form of a “square array” (Wolburg et al., 2011). Moreover, many experiments have demonstrated that AQP4 has a high selectivity of water, which is determined by three specificities on the AQP4 monomer channel. The pores of each monomer are as small as 2.8A, which is conducive to blocking molecules larger than water from passing through (Nagelhus and Ottersen, 2013). The pore region contains an arginine residue that serves to block the entrance of protonated water and other cations (Nagelhus and Ottersen, 2013). Positively charged dipoles in the channel will prevent the flow of protons and cause water molecules to redirect (Nagelhus and Ottersen, 2013).

In the case of cerebral ischemia, AQP4 has a dual effect. In the early stage of stroke, it can quickly cause cerebral edema and brain damage. In the late stage, the up-regulated expression of AQP4 can have a clearing effect on vascular edema (Chen et al., 2021). An animal study in a model of global cerebral ischemia suggests that there was strong neuroprotection and better survival outcome in AQP4 knock-out mice compared with the AQP4 + / + mice after arterial occlusion (Katada et al., 2014). Thus, APQ4 in the astrocytes is a core issue that impacts cerebral edema. It is a very promising treatment for cerebral edema via the modulation of aqueous passage (Rasmussen et al., 2018). Studies also showed that AQP4 is related to post-stroke edema (Zheng et al., 2017; Wang and Parpura, 2018; Kitchen et al., 2020). Therefore, it may become a key point to improve the survival rate of astrocytes for the treatment of cerebral edema.

Aquaporin 4 not only acts as a water channel protein but also has a role in neuroimmunology (Ikeshima-Kataoka, 2016). AQP4-immunoglobulin G (IgG) autoantibody can be used in the differential diagnosis of neuromyelitis optica (NMO) and multiple sclerosis (Jacob et al., 2007). Through exposing astrocytes and oligodendrocytes from primary cultures and rat optic nerves for 24 h to (NMO)-IgG in the absence of the complement, researchers found the complement-independent effect of NMO-IgG/AQP4 antibody on astrocytes, with secondary damage to oligodendrocytes (Marignier et al., 2010). Research shows that AQP4 also plays a protective role in the removal and degradation of beta-amyloid protein (Aβ), which is considered to be one of the influence factors of Alzheimer’s disease (AD; Yang et al., 2012). In addition, the cooperation of AQP4 and GLT-1 in astrocytes has a protective impact against glutamate-induced neuronal injury by Aβ (Lan et al., 2016). Therefore, AQP4 in astrocytes may be the molecular target for the treatment of AD.

**FIGURE 1:**
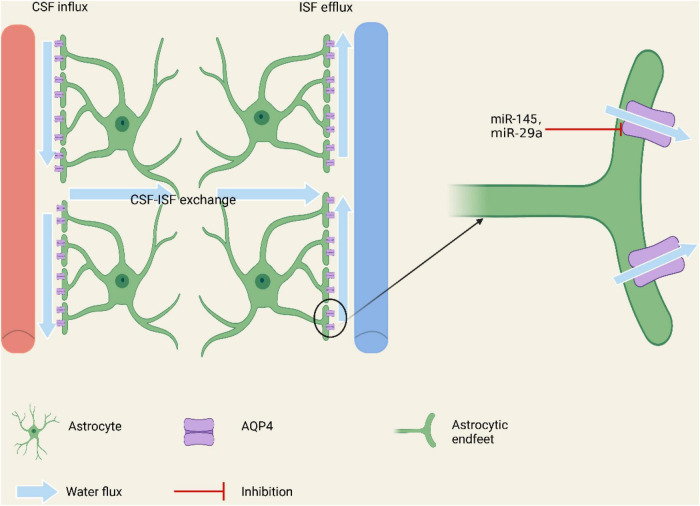
Regulation of miRNAs on AQP4 on the endfeet of astrocytes involving in constituting the brain glymphatic system and the BBB. By regulating the expression of AQP4, miRNAs may affect the water balance, additionally modulating the brain edema in cerebral stoke.

Some scholars artificially produced the condition of oxygen deficiency and glucose starvation to simulate the environment where the stroke occurred (Wang et al., 2020). In an *in vitro* study using primary cultured astrocytes from neonatal rats, the researcher found that the expression of miR-145, which was targeted directly on AQP4, was significantly reduced in astrocytes subjected to oxygen-glucose deprivation (OGD), and over-expression of miR-145 can inhibit OGD-induced apoptosis (Zheng et al., 2017). They also found that miR-145 could inhibit AQP4 expression and AQP4 enhanced astrocyte injury in ischemic stroke, and knocking out of AQP4 lessened the miR-145-mediated protective impact on ischemic injury (Zheng et al., 2017). This reveals that AQP4 plays an important role in astrocyte injury after ischemic stroke, and inhibition of miR-145 expression can increase cerebral edema and worsen the prognosis due to increased expression of AQP4. In another similar study, miR-29a shows the property of alleviating astrocyte apoptosis by inhibiting the AQP4 and improving the survival rate of neurons (Zheng et al., 2019; Figure 1).

## Micro Ribonucleic Acid and Inflammatory Response

**FIGURE 2:**
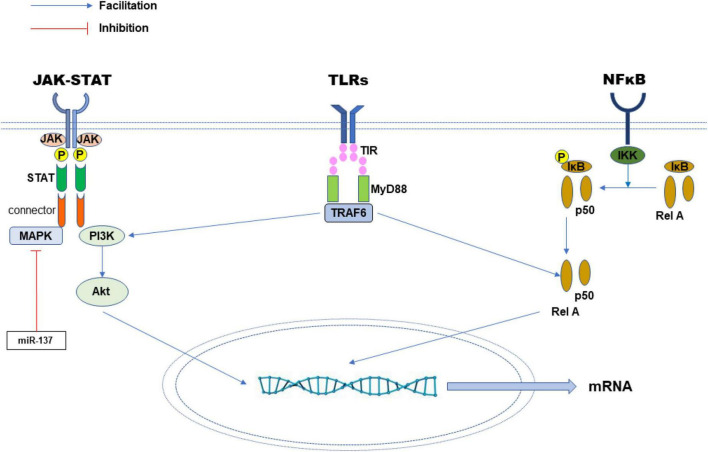
Three classic inflammatory signaling pathways. The miRNA can promote or inhibit the expression of key mediators or proteins in the signaling network, through which the inflammatory response is regulated.

Inflammation is a series of responses to external and internal stimulation. For example, when the body is subjected to a stimulus such as infection or tissue damage, the cells secrete relevant pro-inflammatory and anti-inflammatory substances at the same time to keep inflammatory balance to restore the integrity of structure and function (Medzhitov, 2008). There are generally four steps in which inflammation occurs (Medzhitov, 2010). The first is the signal-inducer of inflammation, whether exogenous or endogenous. The second is the activation of receptors by the inducer to produce specific mediators (Medzhitov, 2008). And the production of the mediators is the third step. The mediators act on specific effectors, such as damaged tissue or cells to change their functional state. In this process, the release of the mediators increases the most after passing through the cascade reaction of the signal pathway (Medzhitov, 2008). Therefore, blocking the release of mediator substances is the most important to interrupt the extension of inflammation (Medzhitov, 2008). In terms of the inflammatory response, three classic pathways are studied repeatedly (Figure 2).

JAK-STAT pathway. JAK and STAT are key parts of many signaling pathways that regulate cell growth, differentiation and survival (Banerjee et al., 2017). The activated JAK protein phosphorylates the receptor and itself. These phosphorylation sites become the binding positions of STAT proteins with SH2 structure and connector proteins. Connector proteins connect the receptors with MAP kinase, PI3K/Akt and other pathways (Steelman et al., 2008). The receptor-bound STAT protein is then phosphorylated by JAK to form a dimer and metastasize into cells to regulate the expression of nuclear regulatory target genes (Villarino et al., 2017). The study found that the JAK-STAT pathway contributes to the occurrence of cancer and inflammation (Banerjee et al., 2017).

NF-κB pathway. NF-κB has long been regarded as a typical signaling pathway to promote inflammation (Lawrence, 2009). The NF-κB/Rel protein in the pathway includes NF-κB2 (p52/p100), NF-κB1 (p50/p105), c-Rel, Rel A (p65) and Rel B. These proteins all form dimer transcription factors (Zhang et al., 2017). The genes they control regulate many biological processes such as congenital immunity, acquired immunity, inflammation and stress response (Lawrence, 2009). In classic pathways, NF-κB/Rel binds to and is suppressed by IκB. Inflammatory factors, lipopolysaccharide (LPS), growth factors and antigen receptors can activate IκB kinase (IKK) complexes including IKKβ, IKKα and NEMO, which will phosphorylate IκB protein and cause IκB protein to be ubiquitinated and degraded in the lysosome (Oeckinghaus et al., 2011). Subsequently, NF-κB is released. Activated NF-κB is further activated by phosphorylation and transferred to the nucleus. NF-κB alone or combined with other transcription factors such as AP-1, Ets and STAT to induce the expression of target genes (Fan et al., 2013). In another NF-κB pathway, the NF-κB2 p100/Rel B complex stays in the cytoplasm in an inactive state (Zhang et al., 2017). The activation of some receptors, such as LTβR, CD40 and BR3 activates kinase NIK, which then activates the IKKα complex, which phosphorylates the carboxyl end amino acids of NF-κB2 p100 (Zhang et al., 2017). After phosphorylation, NF-κB2 p100 is ubiquitinated and degraded by protease bodies to NF-κB2 p52. Finally, an NF-κB2 p52/Rel B complex with transcription activity is formed, which is transferred into the nucleus and to modulate target genes (Karin and Ben-Neriah, 2000).

Toll-like receptors (TLRs) mediated pathway. TLRs play a key role in the inherent immune response by identifying unique pathogen-related molecular characteristics (Moresco et al., 2011). They participate in forming the first line of defense against invasive pathogens and play a significant role in inflammation, immune cell regulation, survival and proliferation. Up to now, 11 members of the TLR family have been found, in which TLR1, 2, 4, 5 and 6 are located on the cell surface, while TLR3, 7, 8 and 9 are on the endoplasmic reticulum and lysosomes (Moresco et al., 2011). The signal transmission of the TLR pathway starts with the intracellular Toll/IL-1 receptor (TIR) domain of the receptor and the connector protein MyD88, which also contains the TIR domain (Brennan and Gilmore, 2018). When stimulated by ligands, MyD88 binds IL-1 receptor-associated kinase (IRK) to TLRs and reacts with each other through the two molecular death domains (Brennan and Gilmore, 2018). IRAK-1 is activated by phosphorylation and then combined with TRAF6, which eventually leads to the activation of JNK and NF-kB (Gilmore and Wolenski, 2012).

After the acute stroke, the production of inflammatory factors of brain tissue increases, where they gathered in the infarction area, resulting in various inflammation injuries *in vivo* (Anrather and Iadecola, 2016). An inflammatory factor is a manifestation of the body to protect the harmful stimulation. The main inflammatory cells in the CNS are microglia/macrophages, leukocytes and mastocytes (Shekhar et al., 2018). The key issue of this inflammatory damage is the destruction of BBB and neurovascular units and may result in systemic inflammatory syndrome, which can lead to the release of cytokines in serum (Yang et al., 2017; Neal et al., 2018).

An *in vitro* study showed astrocytes can be activated into two polarized phenotypes by microglia in the inflammatory response after stroke onset, which includes the neurotoxic A1 phenotype and the neuroprotective A2 phenotype (Liddelow et al., 2017). Microglia activate astrocytes by producing three cytokines: IL-1α, TNFα, and the complement component subunit 1q (C1q) (Liddelow et al., 2017; Xu et al., 2020). The three cytokines can activate A1 astrocytes and subsequently A1 astrocytes contribute to neuronal and oligodendrocyte injury. Thus, the treatment of inflammatory responses of brain tissue to stroke is very important, and a reduction of the number of inflammatory factors can improve patient prognosis and reduce neuronal damage (Liu and Chopp, 2016). It was revealed that some miRNAs secreted by astrocytes can alleviate the release of inflammatory factors at the level of transcription or translation, thereby reducing brain damage and improving the prognosis.

The miR-137 in astrocytes may be involved in regulating the inflammatory response. In an ischemic stroke model using mice experiment, researchers found that miR-137 can target the SRC gene and suppress its expression. Blocking the SRC-mediated MAPK signaling pathway can reduce the production of inflammatory factors such as TNF-α, IL-1, IL-6, and IL-17 in the brain tissue (Tian et al., 2020). This result may suggest that miR-137 regulates inflammatory cytokines and alleviate brain tissue inflammatory response via blocking the SRC gene. One possible mechanism by which astrocytes control miR-137 expression is regulating the expression of lncRNAGAS5. It has been reported that silencing lncRNAGAS5 can upregulate the expression of miR-137 in astrocytes (Jing et al., 2021). In another study, it is reported that by inhibiting the miR-210 gene, the protection of the mice brain in ischemic stroke can be achieved (Huang et al., 2018). Thus, in future research, it is possible to consider high-throughput methods for detecting miR-137 and miR-210 target genes to facilitate better treatment for acute ischemic stroke.

## Micro Ribonucleic Acid and Angiogenesis

The ischemic injury causes the brain tissue to be divided into two different regions. The inner ischemic core and the surrounding ischemic penumbra (Patabendige et al., 2021). In the core area of ischemia, brain cells have died due to a lethal lack of glucose and oxygen, for the blood and energy supply have been cut off resulting in permanent damage (Patabendige et al., 2021). The cells in the penumbra suffer from a non-fatal shortage of blood supply, and thus the injury is reversible to some extent. If the blood and oxygen perfusion can be achieved in time, most cells and tissue in the penumbra are possibly being rescued (Manuel et al., 2017). Angiogenesis is a gradual process involving increased vascular permeability, degradation of surrounding matrix, proliferation and migration of endothelial cells, and stabilization of newly formed microvessels (Viallard and Larrivée, 2017). Therefore, for the treatment of ischemic disease, the ideal regulation would be the expression of vascular endothelial growth factors (VEGF) in response to hypoxia/ischemia (Heydari et al., 2020). Both neurons and astrocytes can express VEGF (Shen et al., 2008). Blood vessel regeneration refers to the formation of new blood vessels in an existing blood vessel, usually under hypoxia conditions and the regulation of Hypoxia-inducible factor-1 (HIF; Patabendige et al., 2021). The mammalian VEGF family consists of five members, namely VEGF–A, –B, –C, –D, and placental growth factor (PGF). Among them, VEGF-A has received the most attention. VEGF-A is pro-angiogenic and neuroprotective (Jing et al., 2021). Hypoxia-inducible factor-1 (HIF-1) is a key transcription factor in the hypoxic brain. It consists of an inducible subunit HIF-1α and a constitutive subunit HIF-1β. HIF-1α is an important activator of VEGF-A gene expression (Geiseler and Morland, 2018).

Experiments detecting the expression levels of angiogenesis-associated miRNAs in acute ischemic stroke patients, middle cerebral artery occlusion (MCAO) rats, and oxygen-glucose deprivation/reoxygenation (OGD/R) human umbilical vein endothelial cells (HUVECs; Du et al., 2019) have shown that the miR-191 expressed in astrocytes can inhibit the production of blood vessels by inhibiting the key regulatory factor Vezf1 (Du et al., 2019). It is a zinc finger transcription factor that is specifically expressed in endothelial cells. Knockout of the miR-191 gene can promote the production of blood vessels in mice. Manuel et al. reported that miR-191 inhibited vascular production by targeting Vezf1, causing the facilitation of ischemic brain injury (Du et al., 2019). Therefore, miR-191 is expected to be a valid biomarker and treatment target for acute stroke (Du et al., 2019).

In the experiment using the OGD cellular models, the changes of nerve function and angiogenesis in rats were observed (Deng et al., 2020). The influencer of transcription factors, miR-130a also shows the potential to inhibit blood vessel generation after stroke. And the results show that the miR-130a inhibits the expression of *XIAP* genes, thereby aggravating cerebral ischemia, and the inhibition of the expression of miR-130a can promote brain-derived growth factor (BNGF) expression after brain injury (Deng et al., 2020). The BNGF is a kind of neurotrophin and has neuroprotective effects in cerebral ischemia in MCAO rats (Deng et al., 2020). Another study using the MACO rat model also reported that inhibition of miR-130a can improve new vessels in brain tissue, and this angiogenesis role of miR-130 works by regulating X-linked inhibitor of apoptosis protein (XIAP; Deng et al., 2020). In addition, both miR-15a/16-1 and VEGF/VEGFR signaling pathways can increase the expression of endothelial nitric oxide synthase (ENOS) to promote vessel regeneration and dilation (Sun P. et al., 2020).

## Micro Ribonucleic Acid and Autophagy

Autophagy is a process in which some cells in lysosomes are degraded (Mizushima and Levine, 2020). At first, the autophagy-related (ATG) genes were found in yeast. The process of autophagy begins with the particles which are wrapped in “autophagosomes” with double-layer or multi-layer membrane vesicles and then transmitted to the lysosome for degradation (Levine and Kroemer, 2019). The autophagy of mammals can be triggered by the lack of growth factors or nutrients, initiating the signals to activate PI3K pathways and downstream kinases, including Akt and mTOR, through receptor tyrosine kinase (Miao et al., 2020). Phosphorylated PTEN negatively regulates PI3K type I (PI3K–I) (Miao et al., 2020). In low-energy situations such as cerebral ischemia, when ATP declines and AMP increases in the cytoplasm, the activation of AMPK inhibits the activity of mTOR and causes autophagy to be activated. The activation of autophagy leads to the reduction of nerve cells, which eventually leads to a poor prognosis for the stroke (Miao et al., 2020).

After ischemic stroke, ischemia is often accompanied by reperfusion injury, alleged ischemia/reperfusion (I/R) injury (Miao et al., 2020). Ischemic postconditioning (IPostC) is a treatment strategy to modulate and alleviate brain injury in the early stage of ischemia (An et al., 2020). One way to optimize the IPostC is to regulate the signaling pathway through miRNAs to reduce neuron autophagy. In autophagy pathways, the treatment often increases the role of PI3K/Akt2 in cerebral I/R injury by activating the phosphorylation of PI3K and Akt2 (Miao et al., 2020). Animal experiments showed that there was a large-scale infarction in the I/R group, and the percentage of cerebral infarction volume in the reperfusion IPostC group of mice significantly decreased. Inhibition of the expression of miR-124 further reduced the percentage of infarction volume, indicating that the downward adjustment of miR-124 from astrocytes is involved in the brain protection of IPostC (Miao et al., 2020). Therefore, miR-124 may reduce cell autophagy induced by brain I/R injury by regulating the signaling pathway of PI3K/Akt2/mTOR (Miao et al., 2020).

## Micro Ribonucleic Acid and Apoptosis

Cell apoptosis is a common way of cell programmed death, which involves cell renewal, proliferation, and growth (Shi, 2002). The main characteristics of apoptosis are changes in nuclei, such as nuclear condensing, shrinking and disappearance, and in other organelles (Maiuri et al., 2007). The apoptotic cells then form the apoptotic bodies near the cell membrane (Jung et al., 2020). Apoptosis is capable of maintaining a dynamic balance between neonatal cells and aging cells (Liu et al., 2017; Fricker et al., 2018; Obeng, 2021). The Caspase family belongs to cysteine proteases. Caspase in the initial group includes Caspase 2, 8, 9, 10, 11 and 12, which are closely connected to the initiation of apoptotic signaling (Flusberg and Sorger, 2015). Once activated, these enzymes will cut and activate the downstream effect group of Caspases, including Caspase 3, 6 and 7, which achieve apoptosis by cutting the specific location of aspartic acid residues of intracellular proteins (Shi, 2002).

Inhibiting the expression of miR-124 in astrocytes can reduce the apoptosis of neurons *in vivo* and *in vitro* after ischemic stroke (Miao et al., 2020). It is also indicated that the inhibition of the expression of miR-124 reduces the expression of apoptosis-related proteins such as Caspase 3 and Bax, whereas increases the expression of anti-apoptotic protein Bcl-2, both of which can reduce the apoptosis of neurons and improve the infarction area of patients (Miao et al., 2020).

## Summary and Outlook

After the stroke, brain tissue ischemia and hypoxia lead to a variety of pathophysiological changes, and it is difficult to offer effective treatment. In the brain, neurons are non-renewable cells, which means the neurofunction will not be repairable once the neurons have been damaged (Arumugam et al., 2018; Chamorro et al., 2021). Astrocytes are superior in numbers and have become the focus of the researches aiming to use renewable characteristics to change the outcome of stroke (Cekanaviciute and Buckwalter, 2016). In recent years, miRNA has become the hot spot, since it can be stably expressed in astrocytes. During the transcription process, the miRNA is a key regulator of gene expression (Lu and Rothenberg, 2018). The control of astrocytes by inhibiting or activating miRNAs can help to reverse the adverse consequences of cerebral ischemia. Treatment for the targets of genes is the most efficient and accurate, and also provides the development of novel drugs. It has been proven that miRNAs have a relatively small molecular weight and they can readily pass the BBB and exhibit stable expression in peripheral blood (Bai et al., 2021). Since the permeability of BBB will increase after stroke (Abdullahi et al., 2018), the concentration of miRNAs in the blood may be used as an indicator that prompts stroke prognosis, and could also help predict the outcome of post-stroke rehabilitation (Mirzaei et al., 2018). Furthermore, studies are showing that miRNA can also be used as a new target for diagnosing CNS injury (Mirzaei et al., 2018).

## Author Contributions

YZ, LL, and HZ established the work. XL helped to revise the manuscript. FC and TL revised and integrated the manuscript and shared correspondence. All authors contributed to the article and approved the submitted version.

## Conflict of Interest

The authors declare that the research was conducted in the absence of any commercial or financial relationships that could be construed as a potential conflict of interest.

## Publisher’s Note

All claims expressed in this article are solely those of the authors and do not necessarily represent those of their affiliated organizations, or those of the publisher, the editors and the reviewers. Any product that may be evaluated in this article, or claim that may be made by its manufacturer, is not guaranteed or endorsed by the publisher.
